# Role of tannic acid against SARS-cov-2 cell entry by targeting the interface region between S-protein-RBD and human ACE2

**DOI:** 10.3389/fphar.2022.940628

**Published:** 2022-08-08

**Authors:** Xi Chen, Ziyuan Wang, Jing Wang, Yifan Yao, Qian Wang, Jiahao Huang, Xianping Xiang, Yifan Zhou, Yintong Xue, Yan Li, Xiang Gao, Lijun Wang, Ming Chu, Yuedan Wang

**Affiliations:** ^1^ Department of Immunology, School of Basic Medical Sciences, Peking University. NHC Key Laboratory of Medical Immunology, Peking University, Beijing, China; ^2^ State Key Laboratory of Natural and Biomimetic Drugs, School of Pharmaceutical Sciences, Peking University, Beijing, China; ^3^ Peking University Science Park, Taizhou, China

**Keywords:** COVID-19, SARS-cov-2, spike protein, SARS-cov-2-RBD, hACE2, tannic acid, mutant strain

## Abstract

Coronavirus disease 2019 (COVID-19) was caused by a new coronavirus, severe acute respiratory syndrome coronavirus 2 (SARS-CoV-2). SARS-CoV-2 utilizes human angiotensin converting enzyme 2 (hACE2) as the cellular receptor of its spike glycoprotein (SP) to gain entry into cells. Consequently, we focused on the potential of repurposing clinically available drugs to block the binding of SARS-CoV-2 to hACE2 by utilizing a novel artificial-intelligence drug screening approach. Based on the structure of S-RBD and hACE2, the pharmacophore of SARS-CoV-2-receptor-binding-domain (S-RBD) -hACE2 interface was generated and used to screen a library of FDA-approved drugs. A total of 20 drugs were retrieved as S-RBD-hACE2 inhibitors, of which 16 drugs were identified to bind to S-RBD or hACE2. Notably, tannic acid was validated to interfere with the binding of S-RBD to hACE2, thereby inhibited pseudotyped SARS-CoV-2 entry. Experiments involving competitive inhibition revealed that tannic acid competes with S-RBD and hACE2, whereas molecular docking proved that tannic acid interacts with the essential residues of S-RBD and hACE2. Based on the known antiviral activity and our findings, tannic acid might serve as a promising candidate for preventing and treating SARS-CoV-2 infection.

## 1 Introduction

The novel Coronavirus disease 2019 (COVID-19) has garnered global attention owing to its rapid person-to-person spread and fatal outcome. On 11 March 2020, the World Health Organization (WHO) designated COVID-19 a public health emergency of international concern. As of May 2022, over 529 million confirmed COVID-19 cases with an estimated case fatality rate of 1.2% has been documented globally according to World Health Organization Coronavirus Dashboard (https://covid19.who.int/).

The COVID-19 is caused by an enveloped RNA coronavirus, namely Severe Acute Respiratory Syndrome Coronavirus 2 (SARS-CoV-2), which shares about 79.6% identity with SARS-CoV (Gorbalenya et al., 2020; Lu et al., 2020; Wu et al., 2020; Zhou et al., 2020; Zhu et al., 2020). SARS-CoV-2 comprises of enveloped spherical particles of 60–140 nm in diameter with a 30 kb single-stranded RNA (ssRNA) genome (Zhu et al., 2020). The 5′-terminus of the genome encodes an overlapping replicase polyprotein 1 ab (pp1ab) and pp1a, which is subsequently cleaved into 15 non-structural proteins (NSPs) by a 33.8 kDa main protease (M^pro^), including RNA-dependent RNA polymerase (RdRP), which is involved in viral replication and transcription (Hillen et al., 2020; Jin et al., 2020; Zhang et al., 2020). The 3′ terminus encodes structural proteins including envelope, membrane, nucleocapsid and spike glycoprotein (SP) (Lu et al., 2020).

SARS-CoV-2 enters cells via the SP and its cellular receptor, angiotensin converting enzyme 2 (ACE2). The SP is a trimer consisting of an S1 and S2 subunit in each monomer (Letko et al., 2020). The S1 subunit contains one N-terminal domain (NTD) and three C-terminal domains (CTD), of which the CTD1 acts as the receptor-binding domain (RBD) of SARS-CoV-2 (Shang et al., 2020b; Ou et al., 2020). During infection, the SARS-CoV-2-RBD (S-RBD) recognizes human ACE2 (hACE2) on the cell surface, and binds to the peptidase domain of hACE2 for viral attachment, triggering a cascade of events that induces the dissociation of the S1 subunit with hACE2 (Lan et al., 2020; Yan et al., 2020). Moreover, the SP is proteolytically activated at the S1/S2 boundary by multiple human proteases, such as transmembrane protease serine 2 (TMPRSS2), resulting in the shedding of S1 subunit and the transition of S2 subunit from a metastable pre-fusion state to a more stable post-fusion state which is essential for the fusion between cell and viral membranes (Hoffmann et al., 2020; Wrapp et al., 2020). Therefore, the binding of S-RBD to hACE2 is the initial step for SARS-CoV-2 to enter target cells (Shang et al., 2020a; Papageorgiou and Mohsin, 2020).

SARS-CoV-2 SP and hACE2 interaction is a promising target for development of drugs for the treatment of COVID-19. As of July 2021, a total of 1,613,076 variants with mutations in the SARS-CoV-2 genome have been reported, posing a major challenge to the development of vaccines (CNCB-NGDC Members and Partners, 2021). Clinical trials are ongoing for the treatment of COVID-19 with a range of vaccines (Callaway, 2020). Measurement of the efficacy will require long-term randomized, observer-blinded, placebo-controlled clinical trials (Lurie et al., 2020; Thanh Le et al., 2020). Alternatively, drug repositioning has been considered as a practicable strategy for swiftly identifying COVID-19 therapies (Riva et al., 2020). Structure-based virtual screening is one of the most effective approaches for drug discovery (Hall and Ji, 2020). Several antiviral candidates against SARS-CoV-2 have been revealed by screening-based methods such as 1,2,3-triazole-phthalimide derivatives (Holanda et al., 2021). Remarkably, the United States Food and Drug Administration has authorized the nucleoside analogue RdRP inhibitor remdesivir for the treatment of COVID-19 (Beigel et al., 2020; Rubin et al., 2020). However, mortality trials of these repurposed antiviral drugs, including remdesivir, lopinavir, hydroxychloroquine, and interferon beta-1a, have shown little or no effect on COVID-19 patients (Cao et al., 2020; Consortium, 2020). Combinational therapy may help to improve clinical effectiveness and decrease plasma concentrations of medicinal medicines (Kalil et al., 2020; Sheahan et al., 2020). In this study, we focused on the potential of repurposing clinically available medications to block the binding of SARS-CoV-2 to hACE2 utilizing a novel artificial-intelligence drug screening technique, which might hasten the development of innovative therapies for COVID-19.

## 2 Methods

### 2.1 Pseudovirus

The HIV lentivirus pseudotyped with the spike glycoprotein of SARS-CoV-2 that contains a *Luc* reporter gene was provided by Sino Biological Inc. (Beijing, China).

### 2.2 Cells

The HEK293T (ATCC^®^ CRL-3216^™^) cells stably expressing hACE2 (HEK293T-hACE2) were generated according to a published protocol (Riva et al., 2020). Briefly, the HEK293T cells were transduced with hACE2-expressing lentivirus (Sino Biological, Beijing, China), and selected with puromycin (InvivoGen) at 2 μg/ml for 14 days. The puromycin resistant cells were cultured in DMEM medium (Gibco), containing 10% FBS (Gibco), 1 μg/ml puromycin, 50 U/mL penicillin and 50 μg/ml streptomycin.

### 2.3 Pharmacophore model generation

The X-ray crystal structure of the S-RBD-hACE2 complex (PDB ID: 6M0J) (Lan et al., 2020) with a high resolution was obtained from the Protein Data Bank (PDB) database and prepared using the Prepare Protein and Minimization protocols of the Discovery Studio 2020 (DS; BIOVIA-Dassault Systèmes, San Diego, United States) (Chu et al., 2018b). Based on artificial-intelligence algorithm, the pharmacophore generation protocol of the Drug Target Space (DTS; EGR Health, Beijing, China) was applied to generate the most representative features of the S-RBD-hACE2 interface (Chu et al., 2018a).

### 2.4 Virtual screening

The FDA-approved Drug Library (TargetMol; L4200) that comprised a total of 1,364 drugs was subjected to pharmacophore-based virtual screening. All these drugs were first optimized using the Prepare Ligand module in DS which generated 100 conformations for screening. Subsequently, the pharmacophore of the S-RBD-ACE2 interface was used to retrieve the drugs by employing the virtual screening module in DTS (Chu et al., 2018a).

### 2.5 Surface plasmon resonance analysis

The recombinant S-RBD (Sino Biological, Beijing, China) and hACE2 protein (Sino Biological, Beijing, China) were used for surface plasmon resonance (SPR) analysis using a Biacore 8K instrument (Biacore, Uppsala, Sweden) as previously described (Chu et al., 2018a; Chu et al., 2018b). Each target was immobilized onto flow cells in a CM5 sensor chip (GE Healthcare) via the amine-coupling method. Briefly, S-RBD was diluted in 10 mM pH 5.5 acetate to 20 µg/ml, while hACE2 was diluted in 10 mM pH 4.5 acetate to 20 µg/ml. Then the protein solutions were injected individually on the carboxyl modified sensor surface to form amine bonds. Both S-RBD and hACE2 immobilized levels were about 10,000 RU. Binding analyses were carried out at 25 °C and a flow rate of 30 μl min^−1^. The retrieved drugs in a running buffer (1×PBS, 0.05% Tween 20 and 5% dimethyl sulfoxide, pH 7.4) were run over each target at gradient concentrations as indicated. An empty flow cell, without any immobilized protein, was used as a deducted reference. The binding curves were analyzed using a kinetic binding model supplied with Biacore Evaluation Software (GE Healthcare).

### 2.6 Competition binding experiment

For competition binding experiment, hACE2 protein was immobilized on the CM5 sensor chip via the amine-coupling method. The immobilized level was 5,000 RU. First, tannic acid was injected for pre-incubation of hACE2 protein. Then, 40 nM of S-RBD was injected to assess the association with hACE2 in the presence of tannic acid. The blocking efficacy was evaluated by comparison of response units with and without tannic acid incubation.

### 2.7 Competitive inhibition experiment

The SARS-CoV-2 inhibitor screening kit (Sino Biological) was applied for the screening of SARS-CoV-2 inhibitors. A 96-well plate was pre-coated with the S-RBD-mFc recombinant protein. After washings, the wells were incubated with 100 μl hACE2-His-Tag and 100 μl inhibitors at gradient concentrations ranging from 160 μM to 0.024 μM, for 1 h at room temperature. 100 μl of anti-His-Tag-HRP was added after washings and incubated for 1 h at room temperature. 100 μl substrate solution was added and incubated for 15 min at room temperature. 50 μl stop solution was added. The plate was read at 450 nm.

### 2.8 Pseudotype-based neutralization assay

According to a published protocol, the HEK293T-hACE2 cells were treated with each drug at gradient concentrations ranging from 80 μM to 0.012 μM for 1 h, and inoculated with the pseudotyped SARS-CoV-2 in the presence of each drug for 2 h (Riva et al., 2020). Then, the cells were replaced with fresh medium and cultured for 16 h. The activity of firefly luciferase was measured using luciferase assay (Promega) for quantitative determination of infected cells.

### 2.9 Molecular simulation

Molecular simulation was performed as previously described (Chu et al., 2018a). Briefly, the crystallographic structure of S-RBD and hACE2 (PDB ID: 6M0J) (Lan et al., 2020) with a high resolution were prepared using the Prepare Protein and Minimization module of DS. Solvent environment was applied in accordance Chemistry at HARvard Macromolecular Mechanics (CHARMM) force field (Brooks et al., 1983) in this process. The binding site of each protein was defined based on the most representative features of the S-RBD-hACE2 interface. Tannic acid was docked into the binding site of S-RBD and hACE2 using the molecular docking module in DTS. The selected poses were subjected to 100 ns molecular dynamics (MD) simulations using a standardized MD protocol through Pipeline Pilot (PP). The MD trajectories were used to calculate accurate binding affinities between ligand and protein targets. The stability of the complex was analyzed and confirmed by plotting root mean square deviation (RMSD) (Chu et al., 2018a; Chu et al., 2018b). The Poisson Boltzmann with non-polar Surface Area (PBSA) model was used to account for the influence of the solvent in binding free-energy calculation. The S-RBD mutants (B.1.1.7 United Kingdom , B.1.1.28/P.1 Brazil, B.1.351 South Aferica and BA.2 South Africa varant) structure were all downloaded from Protein Data Bank (PDB ID: 7LWS, 7LYN, 7EKC, 7U0N) (Gobeil et al., 2021; Han et al., 2021; Geng et al., 2022).

### 2.10 Mutant analysis

The mutation rate of these essential amino acid was collected from the National Genomics Data Center (NGDC) (https://ngdc.cncb.ac.cn/ncov).

## 3 Results

### 3.1 Pharmacophore modeling of SARS-cov-2-rbd-hace2 interface

**FIGURE 1:**
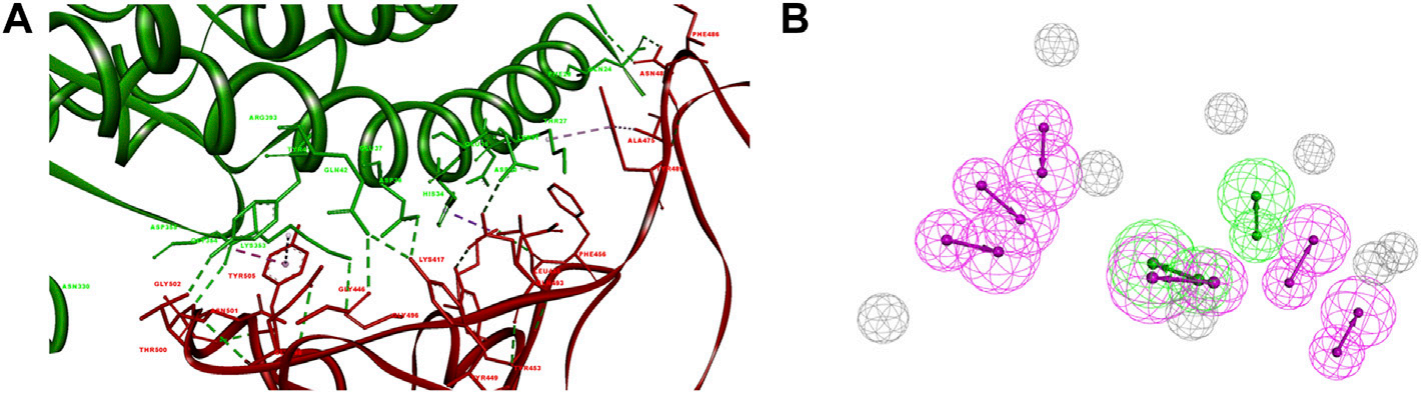
Pharmacophore of SARS-CoV-2-RBD-hACE2 interface. **(A)** The interaction of S-RBD with hACE2. The secondary structural elements were depicted as ribbons (coils, α-helices; arrows, β-sheets). The contacting residues are shown as sticks at the S-RBD-hACE2 interface. The S-RBD and hACE2 were colored in red and green, respectively. The electrostatic interaction was shown as dashed lines with π-π, π-alkyl, and hydrogen bonds colored purple, pink, and green, respectively. **(B)** The pharmacophore features of S-RBD-hACE2 interface. The colored spheres identify the position and the type of binding features (hydrogen bond donor, magenta; hydrogen bond acceptor, green).

Riva et al. have revealed that in the structure of the S-RBD-hACE2 complex (PDB ID: 6M0J), residues Leu455, Phe486, Gln493, and Asn501 are essential for hACE2 binding. The Leu455 interacts with the Asp30, Lys31, and His34 of hACE2, while the Phe486 interacts with the Gln24, Leu79, Met82 and Tyr83 of hACE2, the Gln493 interacts with the Lys31, His34, and Glu35 of hACE2, and the Asn501 interacts with the Tyr41, Lys353, Gly354 and Asp355 of hACE2, respectively (Figure 1A) (Riva et al., 2020).

The pharmacophore was generated using an artificial-intelligence modeling technique, based on the most representative features of the S-RBD-ACE2 interface (Figure 1B). The model was composed of eight features, including two hydrogen-bond acceptor features and six hydrogen-bond donor features that represented the key pharmacophore blocking the binding of S-RBD to hACE2. The two hydrogen-bond acceptor features corresponded to Asn501 and Tyr505 in S-RBD, Glu37 and Tyr41 in hACE2, respectively. Meanwhile the six hydrogen-bond donor features corresponded to Gly446, Tyr449, Leu486, Gln493, Thr500, and Tyr505 in S-RBD, Gln24, Tyr41, Gln42, Glu35 and Arg393 in hACE2, respectively.

### 3.2 Screening of SARS-cov-2-rbd-hace2 inhibitors

The pharmacophore of the S-RBD-hACE2 interface was used as a query to screen the FDA-approved Drug Library with 1,364 drugs. According to the Fit Value greater than 1.0, a total of 20 drugs mapping onto the pharmacophore model were retrieved as S-RBD-hACE2 inhibitors (Table 1). The retrieved inhibitors were well matched to the pharmacophore model of SARS- RBD-hACE2 interface (Supplementary Figure S1).

**TABLE 1:** Virtual screening of SARS-CoV-2-RBD-hACE2 inhibitors.

Number	Drugs	Cas	Fit value	S-RBD	ACE2
				*K*_D_ (µM)	*K*_D_ (µM)
1	Tannic acid	1401–55–4	3.29979	0.179	8.55
2	Nystatin	1400–61–9	3.14239	215.00	NA
3	Sodium aescinate	20977–05–3	3.05158	16.80	NA
4	Teichomycin	61036–62–2	3.00302	47.90	250.00
5	Aescin	6805–41–0	2.98175	3.74	105.00
6	Ginsenoside Rc	11021–14–0	2.76532	79.80	NA
7	Amphotericin B	1397–89–3	2.63099	1.01	9.49
8	Acarbose	56180–94–0	2.58395	NA	NA
9	Geniposide	24512–63–8	2.48703	54.90	NA
10	Vancomycin HCL	1404–93–9	2.24479	NA	NA
11	Mevastatin	73573–88–3	1.95933	273.00	NA
12	Ambroxol	18683–91–5	1.95232	21.20	NA
13	Dapagliflozin	461432–26–8	1.90738	NA	NA
14	Canagliflozin	842133–18–0	1.90689	23.10	NA
15	Simvastatin	79902–63–9	1.78273	113.00	35.80
16	Cytomel	55–06–1	1.56893	16.40	41.70
17	Sodium Picosulfate	10040–45–6	1.45561	16.00	45.40
18	Fasiglifam	1000413–72–8	1.00000	42.20	10.50
19	Pseudolaric acid B	82508–31–4	1.00000	NA	53.80
20	Thyroxine	51–48–9	1.00000	NA	NA

*NA, not applicable.

**FIGURE 2:**
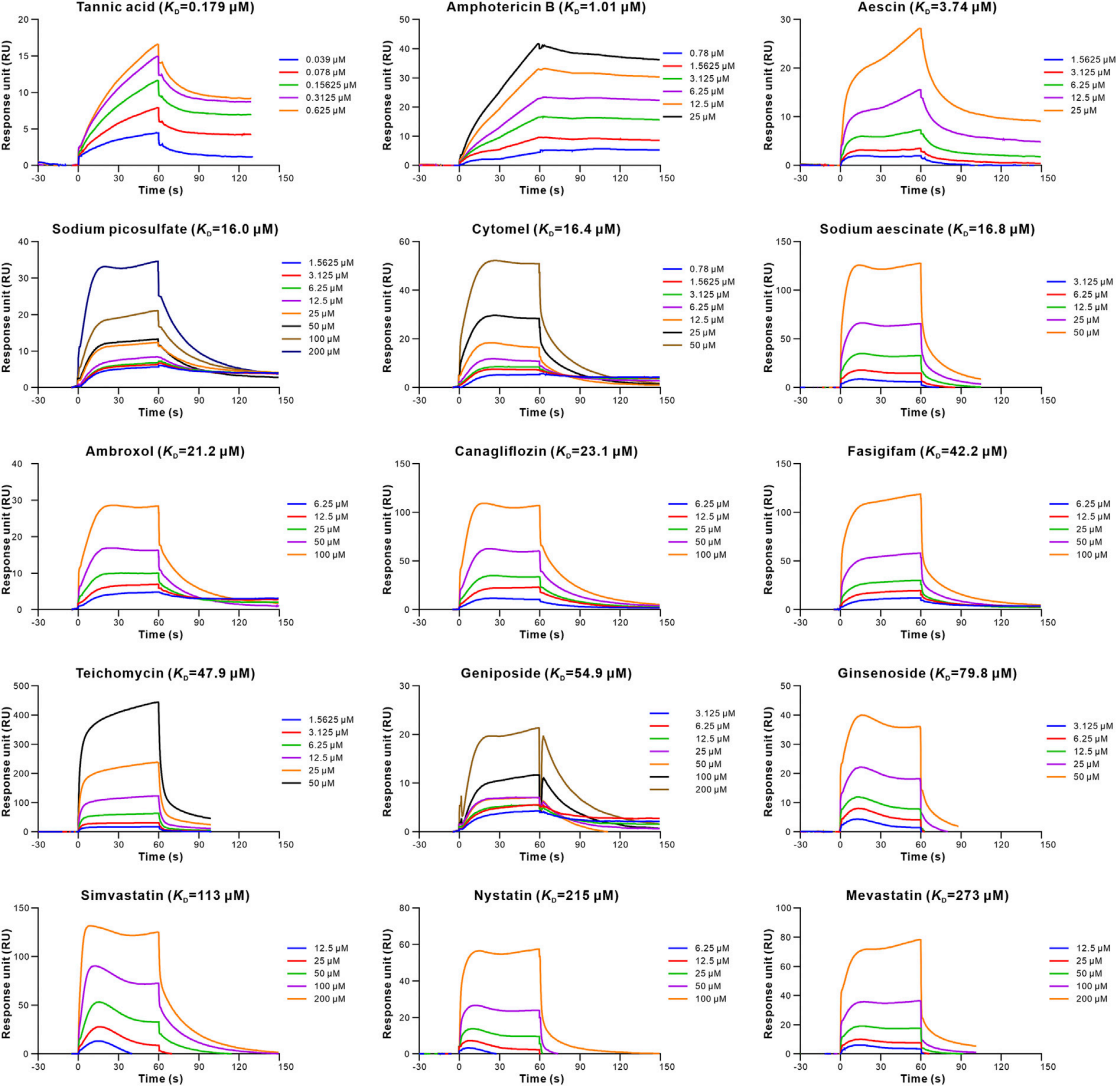
Binding of the retrieved drugs to SARS-CoV-2-RBD. SPR analysis was performed to assess the retrieved drugs binding to S-RBD at gradient concentrations. The binding curves were analyzed using a kinetic analysis supplied with Biacore Evaluation Software. Data are presented as response units (RU) over time (s). The *K*_D_ were calculated by using a 1:1 binding model.

**FIGURE 3:**
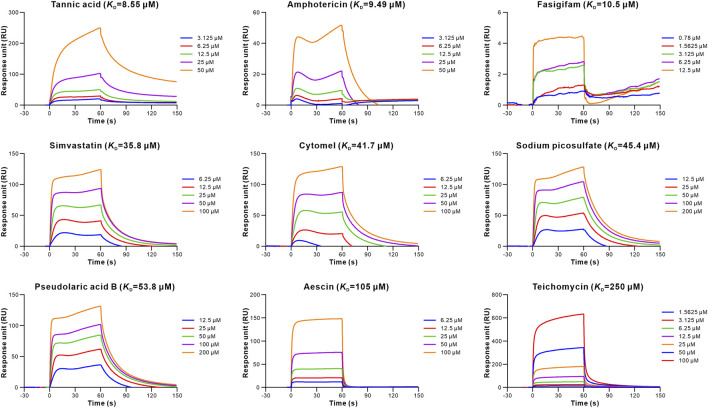
Binding of the retrieved drugs to hACE2. SPR analysis was performed to assess the retrieved drugs binding to hACE2 at gradient concentrations. The binding curves were analyzed using a kinetic analysis supplied with Biacore Evaluation Software. Data are presented as RU over time (s). The *K*_D_ were calculated using a 1:1 binding model.

To evaluate the retrieved inhibitors against the S-RBD-hACE2, surface plasmon resonance (SPR) was used to determine the binding affinities of the 20 retrieved drugs to S-RBD and hACE2. The results showed that 15 drugs bound to S-RBD, with the equilibrium dissociation constants (*K*_D_) of tannic acid being 0.179 μM (Table 1; Figure 2). In addition, nine retrieved drugs bound to the hACE2, with the *K*_D_ of tannic acid being 8.55 μM (Table 1; Figure 3). It is noted that the dissociation rate constant (*K*_d_) between tannic acid and S-RBD was 7.31 × 10^–3^ s^−1^, which was slower than the *K*_d_ between tannic acid and hACE2 (1.05 × 10^–2^ s^−1^). Collectively, 16 drugs were found to bind to the S-RBD or hACE2, with eight drugs targeting both S-RBD and hACE2.

### 3.3 Identification of SARS-cov-2-rbd-hace2 inhibitors

**FIGURE 4:**
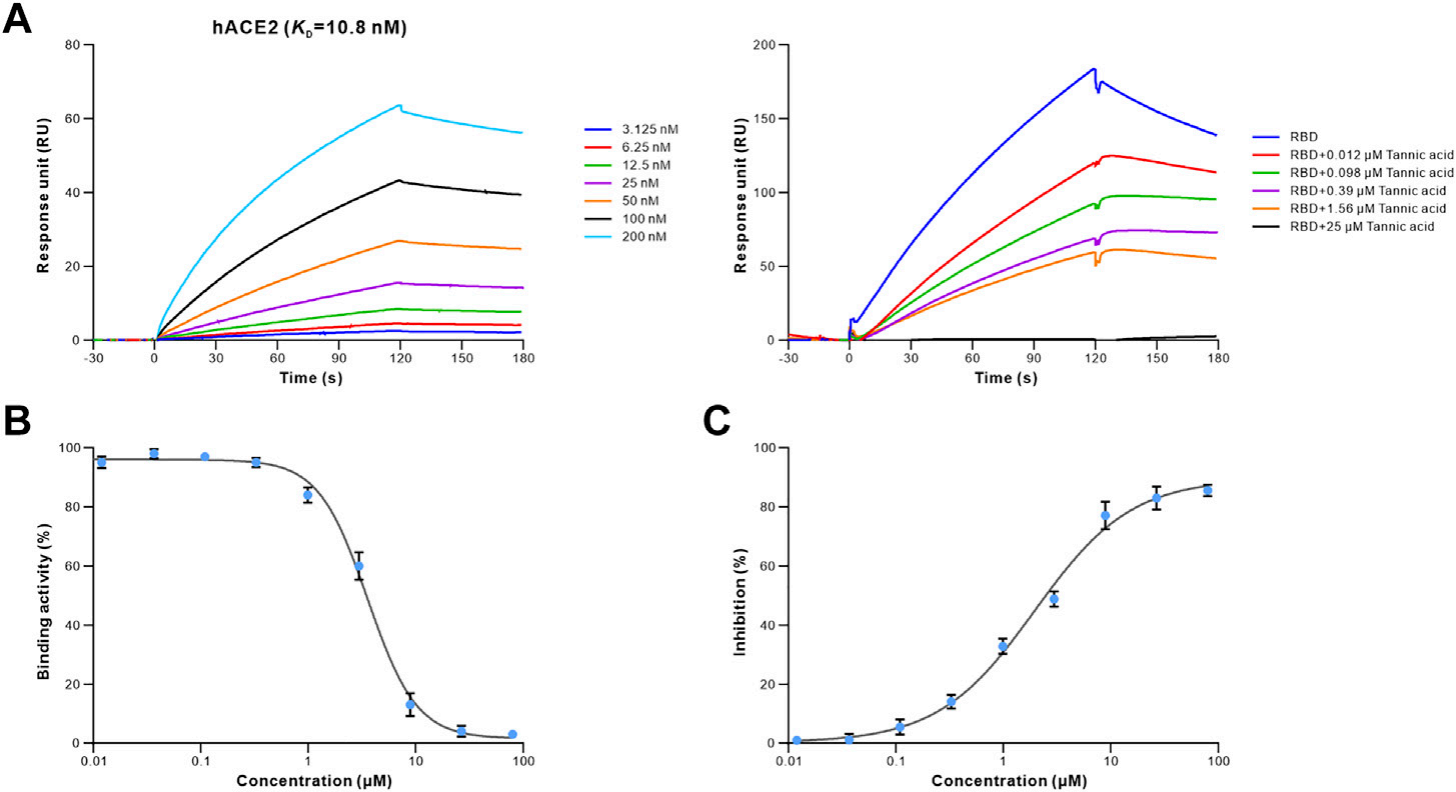
Antiviral activity of tannic acid against SARS-CoV-2. The binding activity of S-RBD to hACE2 in the presence of increasing concentrations of tannic acid. **(A)** SPR analysis was performed to assess the S-RBD binding to hACE2 (left). 40 nM of S-RBD was used in the competitive SPR experiment at gradient concentrations ranging from 25 μM to 0.012 μM (right). The binding curves were analyzed using a kinetic analysis supplied with Biacore Evaluation Software. Data are presented as RU over time (s). The *K*_D_ were calculated using a 1:1 binding model. **(B)** Competitive ELISA was performed to analyze the inhibition activity of tannic acid on S-RBD binding to hACE2. The dose-response curve for IC_50_ value was determined by nonlinear regression. **(C)** Antiviral activity of tannic acid against pseudotyped SARS-CoV-2 in HEK293T-hACE2 cells. Pseudotyped SARS-CoV-2 was used to infect the HEK293T-hACE2 cells pre-incubated with tannic acid at gradient concentration ranging from 80 μM to 0.004 μM. The luciferase activities in cell lysates were determined at 48 h post transduction to calculate infection (%) relative to non-treated control. Data were shown as mean ± SEM of a representative experiment.

To determine whether the 16 retrieved drugs impede the binding of S-RBD to hACE2, we performed competitive ELISA using a SARS-CoV-2 inhibitor screening kit. Remarkably, only tannic acid out of the 16 drugs prevented S-RBD from binding to hACE2 at a concentration of 1 μM (Supplementary Figure S2). Furthermore, the competition binding experiment revealed that tannic acid interfered with the binding of the S-RBD to hACE2 in a dose-dependent manner (Figure 4A). Using the competitive inhibition ELISA technique, the half maximal inhibitory concentration (IC_50_) of tannic acid was determined to be 3.580 μM (Figure 4B). To evaluate the antiviral activity of tannic acid on viral entry, the HEK293T-hACE2 cells were treated with tannic acid at gradient concentrations ranging from 80 μM to 0.004 μM. As expected, tannic acid inhibited infection of HEK293T-hACE2 cells by pseudotyped SARS-CoV-2 with an IC_50_ of 1.959 μM (Figure 4B).

### 3.4 Binding characterization of SARS-cov-2-rbd-hace2 inhibitors

**FIGURE 5:**
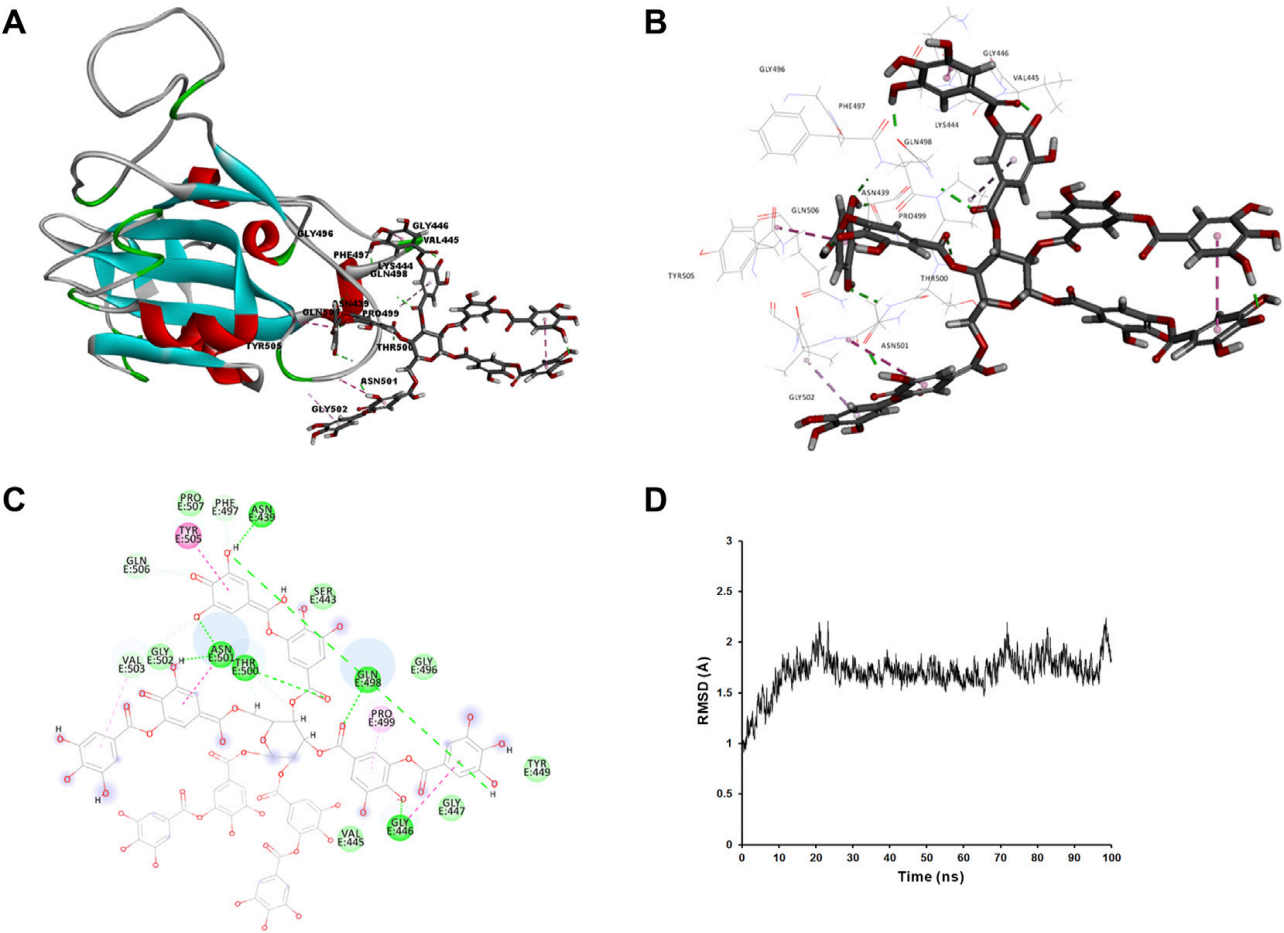
Molecular interaction of tannic acid with SARS-CoV-2-RBD. Tannic acid and key residues were shown as sticks with carbon, oxygen and nitrogen colored gray, red and blue, respectively. Electrostatic interaction was shown as dashed lines with π-π, π-alkyl, and hydrogen bonds colored purple, pink, and green, respectively. **(A)** Binding feature of tannic acid with S-RBD. Secondary structural elements are depicted as ribbons (coils, α-helices; arrows, β-sheets). Color is based on secondary structures (α-helices, red; β-sheets, sky-blue; loops, green). **(B)** Molecular interaction of tannic acid with the residues of S-RBD. The residues were shown in sticks and labeled in black. **(C)** Molecular interaction schemes of tannic acid with the residues of S-RBD. **(D)** MD simulation of tannic acid in complex with S-RBD. The selected pose was subjected to 100 ns MD simulations using a standardized MD protocol through Pipeline Pilot. The stabilities were analyzed by RMSD vs time (ns).

**FIGURE 6:**
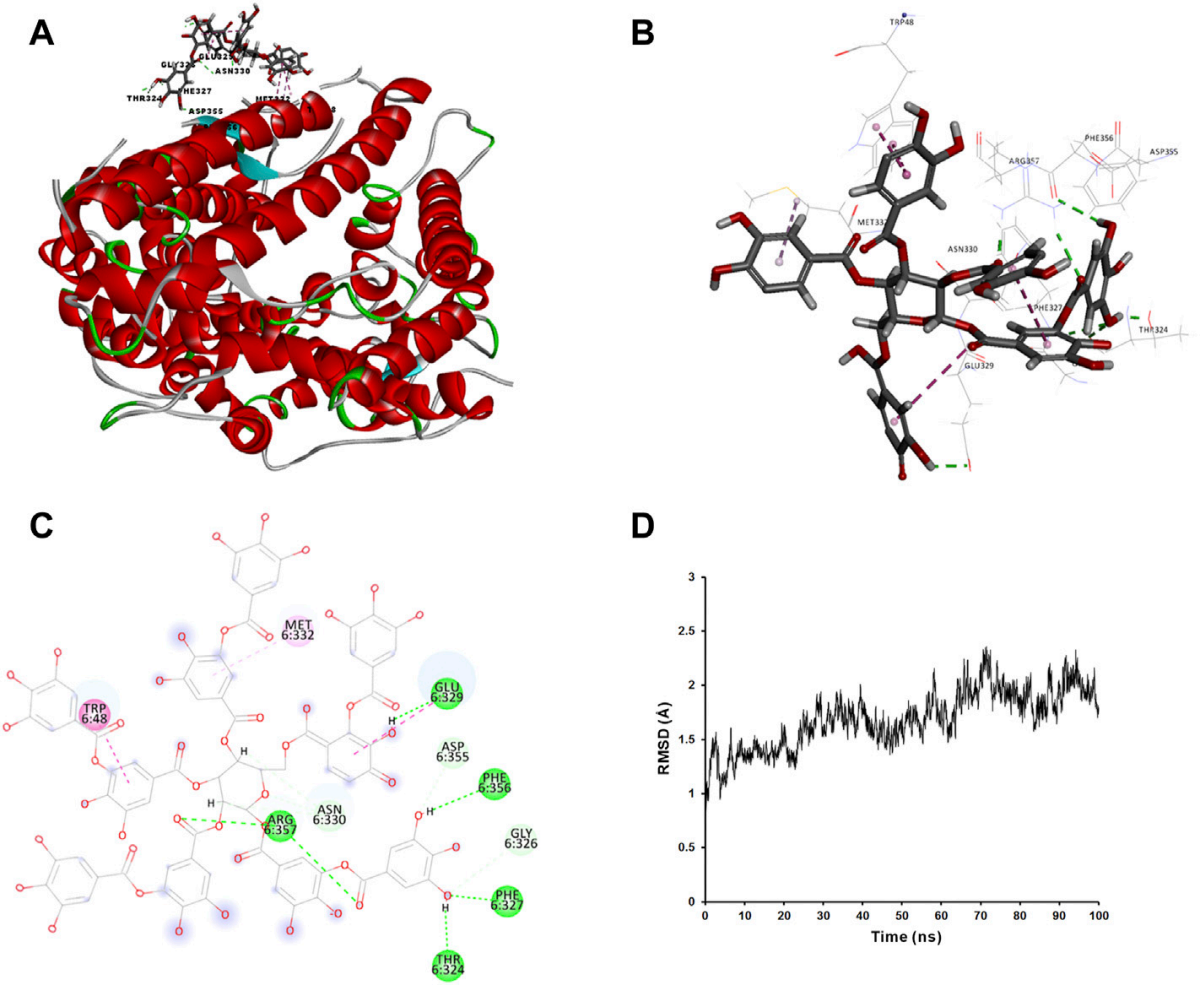
Molecular interaction of tannic acid with hACE2. Tannic acid and key residues were shown as sticks with carbon, oxygen and nitrogen colored gray, red and blue, respectively. Electrostatic interaction was shown as dashed lines with π-π, π-alkyl, and hydrogen bonds colored purple, pink, and green, respectively. **(A)** Binding feature of tannic acid with hACE2. Secondary structural elements are depicted as ribbons (coils, α-helices; arrows, β-sheets). Color is based on secondary structures (α-helices, red; β-sheets, sky-blue; loops, green). **(B)** Molecular interaction of tannic acid with the residues of hACE2. The residues were shown in sticks and labeled in black. **(C)** Molecular interaction schemes of tannic acid with the residues of hACE2. **(D)** MD simulation of tannic acid in complex with hACE2. The selected pose was subjected to 100 ns MD simulations using a standardized MD protocol through Pipeline Pilot. The stabilities were analyzed by RMSD vs time (ns).

We applied molecular docking to analyze the interaction of tannic acid with S-RBD and hACE2 based on the contacting residues on the S-RBD-hACE2 interface. As illustrated in Figure 5 and Figure 6, tannic acid was docked into the defined binding site of S-RBD and hACE2 in a stable state with the binding free-energy of -11.4 kcal mol^−1^ and -27.7 kcal mol^−1^ under PBSA model. Tannic acid interacted with S-RBD residues Gly446, Gln498, Thr500, Asn501, Gly502 and Tyr505, and hACE2 residues Asp355 and Arg357, respectively, which have been demonstrated to be essential for the binding of S-RBD to hACE2 (Figure 5 and Figure 6).

Additionally, we analyzed the mutation rate of the essential amino acids in the tannic-acid-S-RBD interaction (Supplementary Table S2). 10,310,212 SARS-CoV-2 genomes were obtained from the National Genomics Data Center, of which 5,021,029 mutations were inspected (2022–03–29) (CNCB-NGDC Members and Partners, 2021). Nonetheless, the almost identical protein backbone of S-RBD and essential residues in S-RBD binding with tannic acid among origin SARS-CoV-2 and B.1.1.7 United Kingdom, B.1.1.28/P.1 Brazil and B.1.351 South Africa variant allowed tannic acid to share the same binding mode with the original co-crystal structure (Supplementary Figure S4 and Supplementary Table S2). For the newest Omicron BA.2 South Africa strain, the binding conformation of tannic acid to S-RBD altered (Supplementary Figure S4 and Supplementary Table S2) primarily due to the mutation of key amino acids in the interaction interface such as Gln498Arg and Asn501Tyr (Supplementary Figure S3 and Supplementary Table S1), which induced novel electrostatic interactions while weakening original hydrophobic interactions (Supplementary Table S2). These results highlighted the antiviral potential of tannic acid against typical SARS-CoV-2 variants.

## 4 Discussion

The continuing COVID-19 pandemic poses a global hazard to public health. Patients with COVID-19 should receive appropriate therapy for symptom relieve, and those with severe illness should receive optimal supportive care. Given the rapid spread of SARS-CoV-2, improved therapeutic options for COVID-19 are urgently needed. It is noteworthy that severe SARS-CoV-2 infection is a biphasic illness that offers United States therapeutic window to maximize the benefit of antiviral drug treatment (Sundararaj Stanleyraj et al., 2021).

Here, we conducted a high-throughput screening of clinically available drugs against SARS-CoV-2 entry using a novel artificial-intelligence drug screening technique. A total of 20 drugs were retrieved as potent S-RBD-hACE2 inhibitors, of which 16 drugs were identified to bind to S-RBD or hACE2. In addition, eight drugs were able to target both S-RBD and hACE2, with tannic acid having the highest binding affinity. The binding affinity between tannic acid and S-RBD was 47.77 times higher than that between tannic acid and hACE2, indicating that tannic acid tended to bind to S-RBD. Further study indicated that tannic acid interfered with the binding of S-RBD to hACE2 in the clinical therapeutic dose range, and exhibited effective antiviral activity against pseudotyped SARS-CoV-2 entry. Molecular docking and molecular dynamic revealed that tannic acid could block S-RBD from binding to hACE2 by interacting with the essential residues of S-RBD-hACE2 interface, which is also in accordance with a recent research (Haddad et al., 2022). Moreover, it is known that the SARS-CoV-2 spike glycoprotein is broadly mutated, with approximately 90% of the sequence being changed (Bhattacharya et al., 2021). However, tannin still has the ability to bind the S-RBD of B.1.1.7 United States, B.1.1.28/P.1 Brazil and B.1.351 South Africa mutants in the same conformation, as well as has the potential to bind the newest Omicron BA.2 strain. This indicates that tannic acid might have broad antiviral activity against SARS-CoV-2.

Tannic acid is a natural polyphenol found in various grains, fruits, vegetables, herbs and beverages such as tea, coffee and red wine (Kaczmarek, 2020). In traditional medicine, tannic acid has been widely applied as an antidote against different poisons. Nowadays, tannic acid has been topically used to treat cold sores, diaper rash, fever blisters and poison ivy. In addition, tannic acid can also be taken orally to treat bleeding, dysentery, chronic diarrhea, bloody urine, painful joints, persistent coughs. The clinical efficacy of tannic acid is related to its antioxidant, antibacterial and free radicals trapping properties (Skrovankova et al., 2015; Jiang, 2019). Remarkably, increasing evidence indicate that tannic acid exhibits unique antiviral activity against influenza A virus, papillomaviruses, noroviruses, human immunodeficiency virus, hepatitis C virus and herpes simplex virus type 1 and 2, which is related to the inhibition of viral adsorption and entry (Uchiumi et al., 2003; Szymańska et al., 2018; Shirasago et al., 2019). Recent studies suggested that tannic acid suppressed the viral entry by inhibition of SARS-CoV-2 M^pro^ and TMPRSS2 (Coelho et al., 2020; Wang et al., 2020). Consistently, our study demonstrated that tannic acid inhibits SARS-CoV-2 entry through interfering with the binding of S-RBD to hACE2. Comparing with other studies of tannic acid, we have applied both in silico and *in vitro* modelling studies and biological efficacy evaluations. Tannic acid may serve as a promising candidate for the prevention of SARS-CoV-2 infection based on its known pharmacological and safety profiles.

In this research, the pseudotyped SARS-CoV-2 was employed in neutralization experiment. Previous studies have manifested that pseudotype-based neutralization assay is a valid alternative to using the wild-type strain (Hyseni et al., 2020; Schmidt et al., 2020; Riepler et al., 2021), so we anticipated no appreciable differences in the conclusion. Still, more experiments are needed to validate the inhibition ability towards S-RBD-hACE2 interactions of typical SARS-CoV-2 mutants. Furthermore, it is possible that the cell lines used in this study (HEK293T cells) might not fully represent the primary respiratory epithelial cells *in vivo*. Further work is still needed to validate the therapeutic effect of tannic acid in the clinical scenario.

## 5 Conclusion

The rapid spread and frequent mutation of SARS-CoV-2 virus have brought great challenges to global public health. Thus, repurposing of clinical available drugs becomes a promising method to prevent SARS-CoV-2 effectively. In this study, a pharmacophore of S-RBD-hACE2 based on the structure of S-RBD and hACE2 was utilized to screen a library of FDA-approved drugs. A total of 20 drugs were retrieved as potent S-RBD-hACE2 inhibitors, with 16 drugs identified to bind to S-RBD or hACE2 and eight drugs able to target both S-RBD and hACE2 through SPR analysis. Notably, tannic acid showed the highest binding affinity and more tended to bind to S-RBD than hACE2. Tannic acid was validated to inhibited pseudotyped SARS-CoV-2 entry by interfering with the binding of S-RBD to hACE2. Molecular docking proved that tannic acid interacts with the essential residues of RBD and hACE2. Based on the known antiviral activity and our results, tannic acid may serve as a promising candidate for the prevention and treatment against typical subtypes of SARS-CoV-2 infection.

## Data Availability

The original contributions presented in the study are included in the article/Supplementary Material, further inquiries can be directed to the corresponding authors.
