# Supplementary material for: Role of tannic acid against SARS-cov-2 cell entry by targeting the interface region between S-protein-RBD and human ACE2
Source: Front Pharmacol. 2022 Aug 8;13:940628. doi: 10.3389/fphar.2022.940628 (PMC9393390; doi:10.3389/fphar.2022.940628)
Supplement: Supplementary file 1 [file DataSheet1.ZIP › Supplementary_Material.docx]

Supplementary Material

# Supplementary Figures


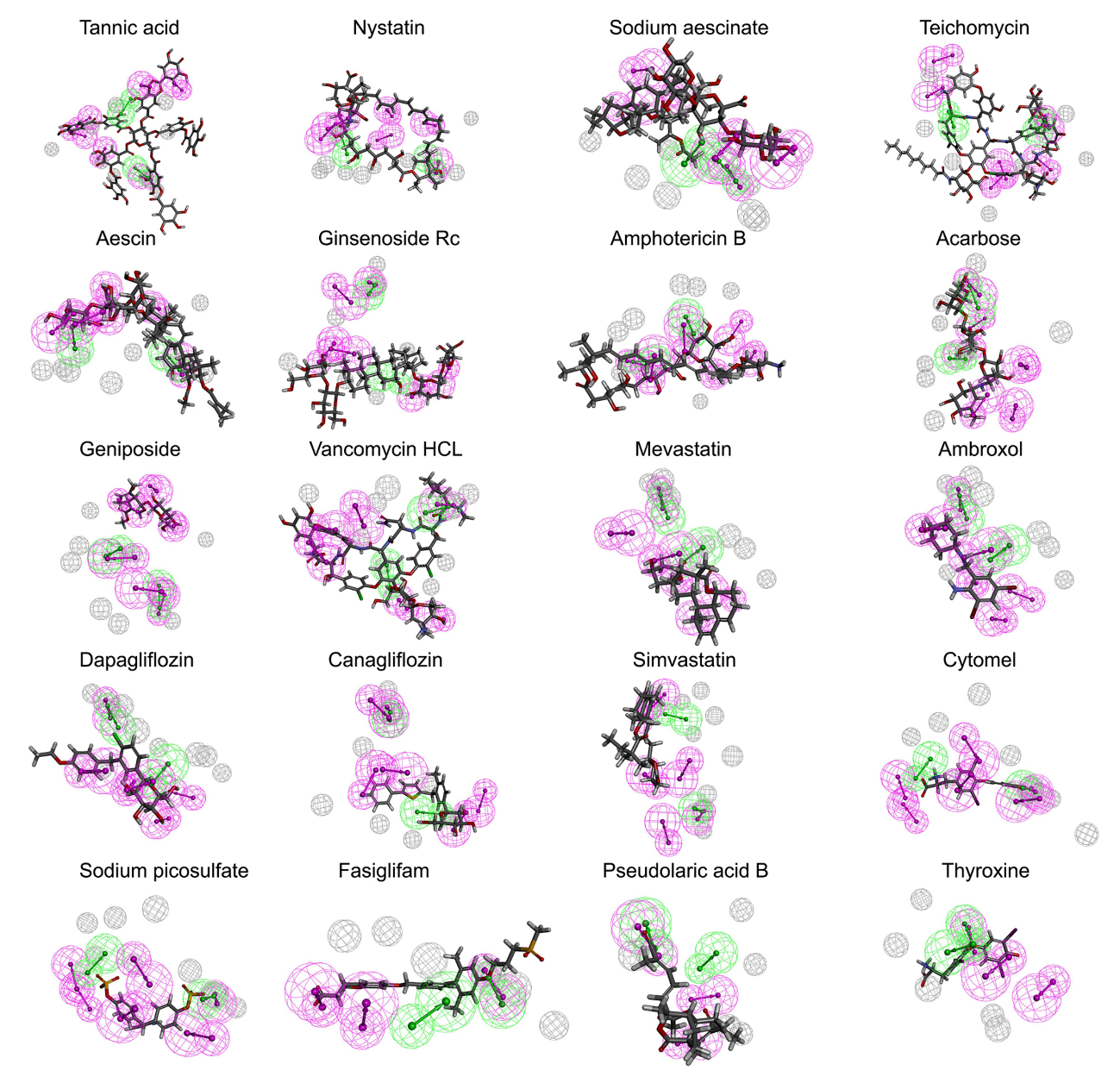


**Figure S1.** The retrieved inhibitors matched to the pharmacophores of SARS-CoV-2 RBD-hACE2 interface. The candidate drugs were shown as sticks with carbon, oxygen and nitrogen colored gray, red and blue, respectively. The colored spheres identify the position and the type of binding features (hydrogen bond donor, magenta; hydrogen bond acceptor, green).


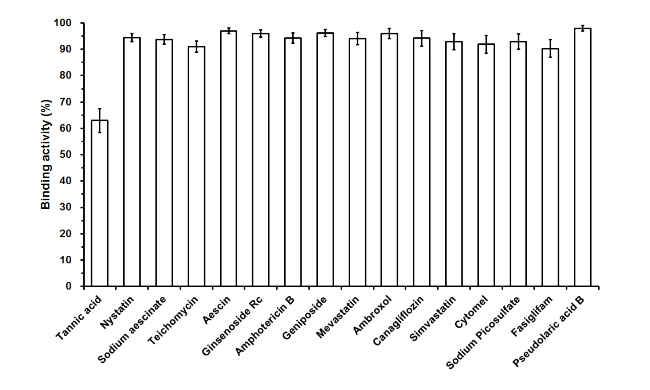


**Figure S2.** Identification of the retrieved inhibitors. Binding activity of SARS-CoV-2 RBD to hACE2 in the presence of 16 retrieved inhibitors at a concentration of 1 μM. The binding activities (%) were calculated in the percent of non-treated control. Data were shown as mean ± SEM of a representative experiment.

**
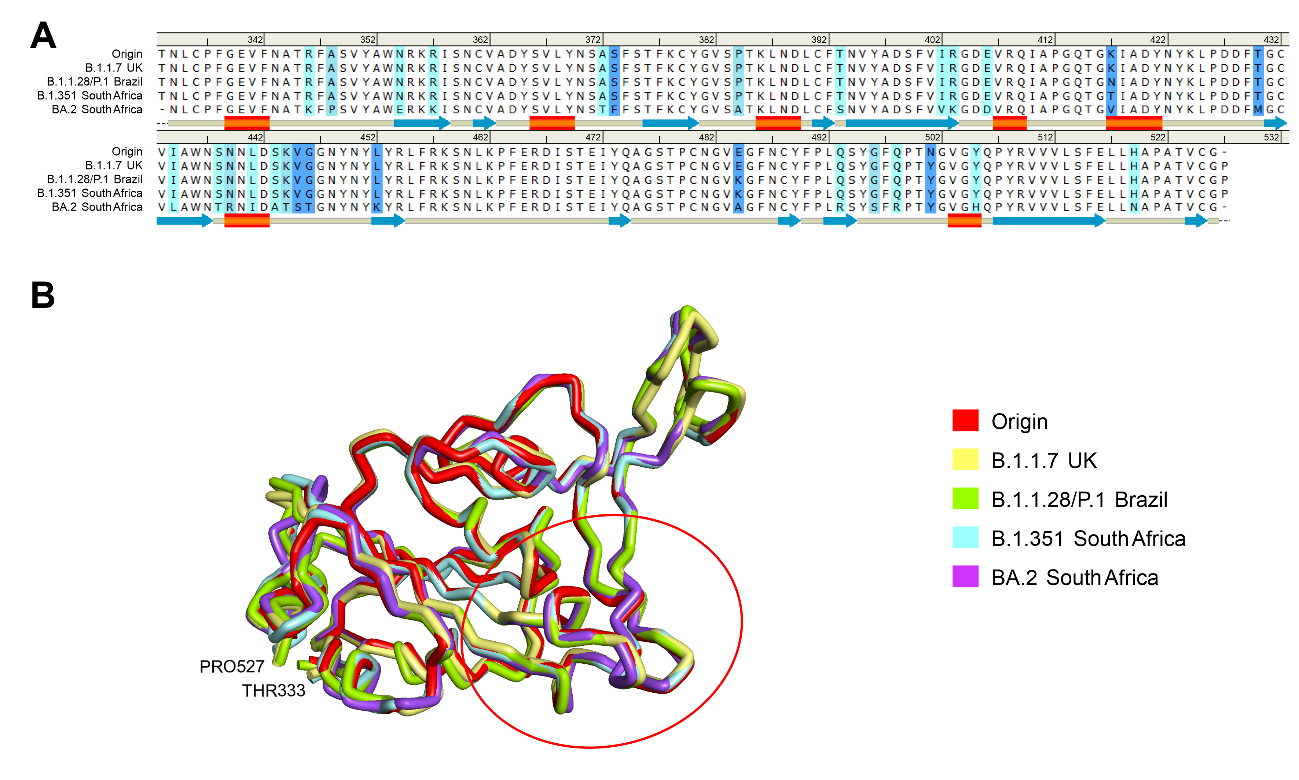
**

**Figure S3.** Overlap of SARS-CoV-2 RBD mutants. (**A**) Amino Acid Sequence of the common SARS-CoV-2 RBD mutants (B.1.1.7 UK, B.1.1.28/P.1 Brazil, B.1.351 South Africa and BA.2 South Africa variant). The blue block indicates the mutation. (**B**) The SARS-CoV-2 RBD mutant structures were illustrated by sticks. The red circle highlights interaction amino acids in these RBD proteins.


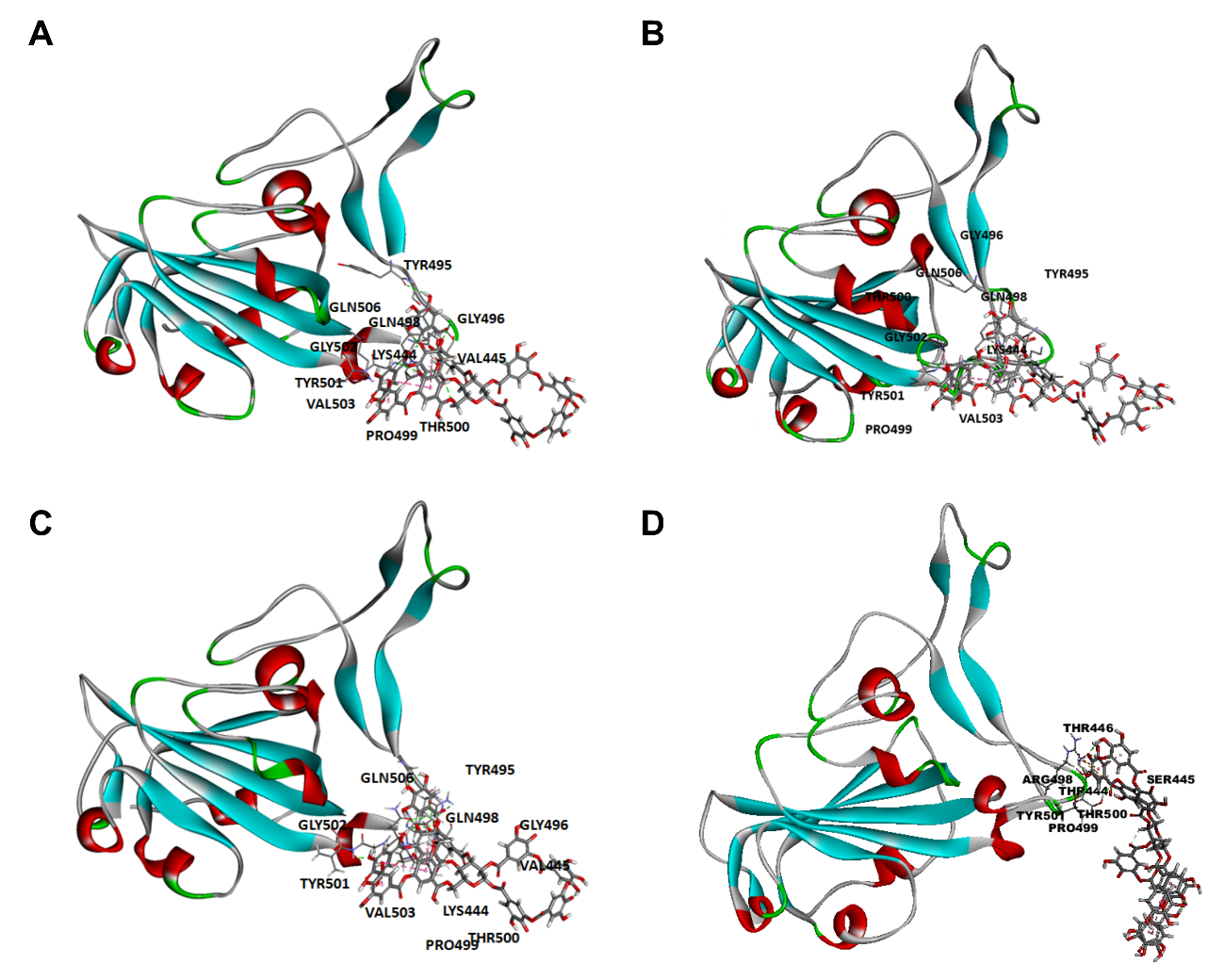


**Figure S4.** Tannic acid binding with typical mutants. (**A**) - (**D**) Docking analysis of tannic acid with common SARS-CoV-2 RBD mutants (B.1.1.7 UK, B.1.1.28/P.1 Brazil, B.1.351 South Africa and BA.2 South Africa variant). Tannic acid and key residues are shown as sticks with carbon, oxygen and nitrogen colored gray, red and blue, respectively. Electrostatic interaction was shown as dashed lines with π-π, π-alkyl, and hydrogen bonds colored purple, pink, and green, respectively. Secondary structural elements are depicted as ribbons (coils, α-helices; arrows, β-sheets). Color is based on secondary structures (α-helices, red; β-sheets, skyblue; loops, green)**.**

# Supplementary Tables

**Table S1.** Mutation rate of the essential amino acids in the interaction of tannic acid SARS-CoV-2 RBD.

| Genomic location | AA mutation | No. of AA with the mutation | AA mutation rate |
| --- | --- | --- | --- |
| 22895 | 445V>L | 5 | 4.84956E-07 |
|  | 445V>I | 164 | 1.59066E-05 |
|  | 445V>F | 186 | 1.80404E-05 |
| 22896 | 445V>G | 7 | 6.78939E-07 |
|  | 445V>D | 5 | 4.84956E-07 |
|  | 445V>A | 310 | 3.00673E-05 |
| 22898 | 446G>C | 2 | 1.93982E-07 |
|  | 446G>R | 194 | 1.88163E-05 |
|  | 446G>S | 729,833 | 0.07078739 |
| 22899 | 446G>A | 66 | 6.40142E-06 |
|  | 446G>D | 163 | 1.58096E-05 |
|  | 446G>V | 6,585 | 0.000638687 |
| 23054 | 498Q>K | 48 | 4.65558E-06 |
|  | 498Q>E | 25 | 2.42478E-06 |
| 23055 | 498Q>L | 8 | 7.7593E-07 |
|  | 498Q>R | 1,143,900 | 0.110948252 |
|  | 498Q>P | 3 | 2.90974E-07 |
| 20356 | 498Q>H | 43 | 4.17062E-06 |
| 23060 | 500T>A | 26 | 2.52177E-06 |
|  | 500T>P | 6 | 5.81947E-07 |
|  | 500T>S | 67 | 6.49841E-06 |
| 23061 | 500T>I | 20 | 1.93982E-06 |
|  | 500T>N | 11 | 1.0669E-06 |
|  | 500T>S | 35 | 3.39469E-06 |
| 23063 | 501N>D | 18 | 1.74584E-06 |
|  | 501N>H | 46 | 4.4616E-06 |
|  | 501N>Y | 2,042,528 | 0.198107275 |
| 23064 | 501N>I | 98 | 9.50514E-06 |
|  | 501N>S | 332 | 3.22011E-05 |
|  | 501N>T | 3,289 | 0.000319004 |
| 23065 | 501N>K | 30 | 2.90974E-06 |

* AA, amino acids.

**Table S2.** Key amino acids of interactions.

| **Interaction** | **Type of interaction** | **Key amino acids** |
| --- | --- | --- |
| S-RBD and ACE2 | Hydrogen bonds | Lys353, Asp38, Met82, Glu35, Lys31 |
|  | Hydrophobic interactions | Asn501, Gln493, Leu455, Ser494, Phe486 |
| S-RBD and tannic acid | Hydrogen bonds | Gly446, Asn439, Thr500, Gln498 |
|  | Hydrophobic interactions | Lys444, Val445, Gly496, Phe497, Pro499, Gly502, Val503, Gln506, Pro507 |
| ACE2 and tannic acid | Hydrogen bonds | Thr324, Phe327, Glu329, Arg357 |
|  | Hydrophobic interactions | Trp48, Asn330, Met332, Arg357 |
| B.1.1.28 (Brazil) S-RBD and tannic acid | Hydrogen bonds | Gly446, Asn439, Thr500, Gln498 |
|  | Hydrophobic interactions | Lys444, Val445, Gly496, Phe497, Pro499, Gly502, Val503, Gln506, Pro507 |
| B.1.1.7 (UK) S-RBD and tannic acid | Hydrogen bonds | Gly446, Asn439, Thr500, Gln498 |
|  | Hydrophobic interactions | Lys444, Val445, Gly496, Phe497, Pro499, Gly502, Val503, Gln506, Pro507 |
| B.1.351 (South Africa) S-RBD and tannic acid | Hydrogen bonds | Gly446, Asn439, Thr500, Gln498 |
|  | Hydrophobic interactions | Lys444, Val445, Gly496, Phe497, Pro499, Gly502, Val503, Gln506, Pro507 |
| BA.2 (South Africa) S-RBD and tannic acid | Hydrogen bonds | Thr444, Ser445, Thr446, Pro499, Thr500, Tyr501 |
|  | Electrostatic | Arg498 |
