# Supplementary material for: Role of tannic acid against SARS-cov-2 cell entry by targeting the interface region between S-protein-RBD and human ACE2
Source: Front Pharmacol. 2022 Aug 8;13:940628. doi: 10.3389/fphar.2022.940628 (PMC9393390; doi:10.3389/fphar.2022.940628)
Supplement: Supplementary file 1 [file DataSheet1.ZIP › Supplementary Material.pdf]

Supplementary Material

1 Supplementary Figures

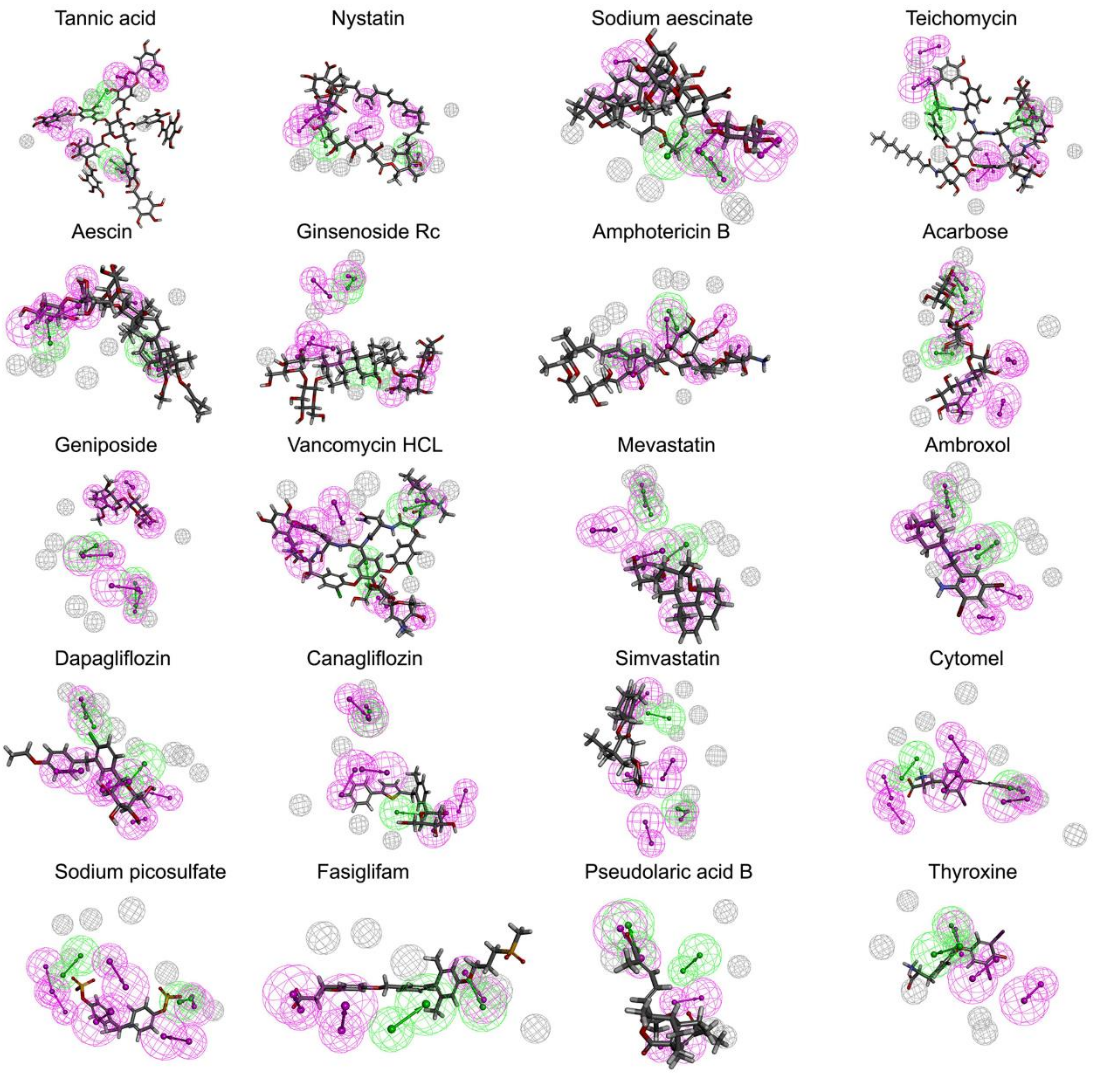

Figure S1. The retrieved inhibitors matched to the pharmacophores of SARS-CoV-2 RBD-hACE2 interface. The candidate drugs were shown as sticks with carbon, oxygen and nitrogen colored gray, red and blue, respectively. The colored spheres identify the position and the type of binding features (hydrogen bond donor, magenta; hydrogen bond acceptor, green).

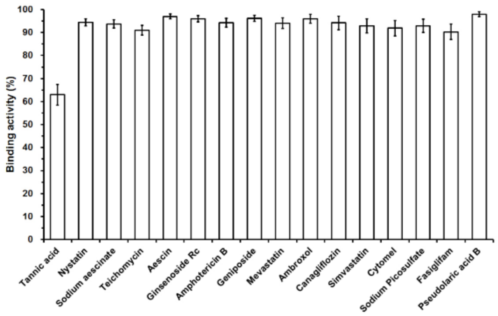

Figure S2. Identification of the retrieved inhibitors. Binding activity of SARS-CoV-2 RBD to hACE2 in the presence of 16 retrieved inhibitors at a concentration of 1 μM. The binding activities (%) were calculated in the percent of non-treated control. Data were shown as mean ± SEM of a representative experiment.

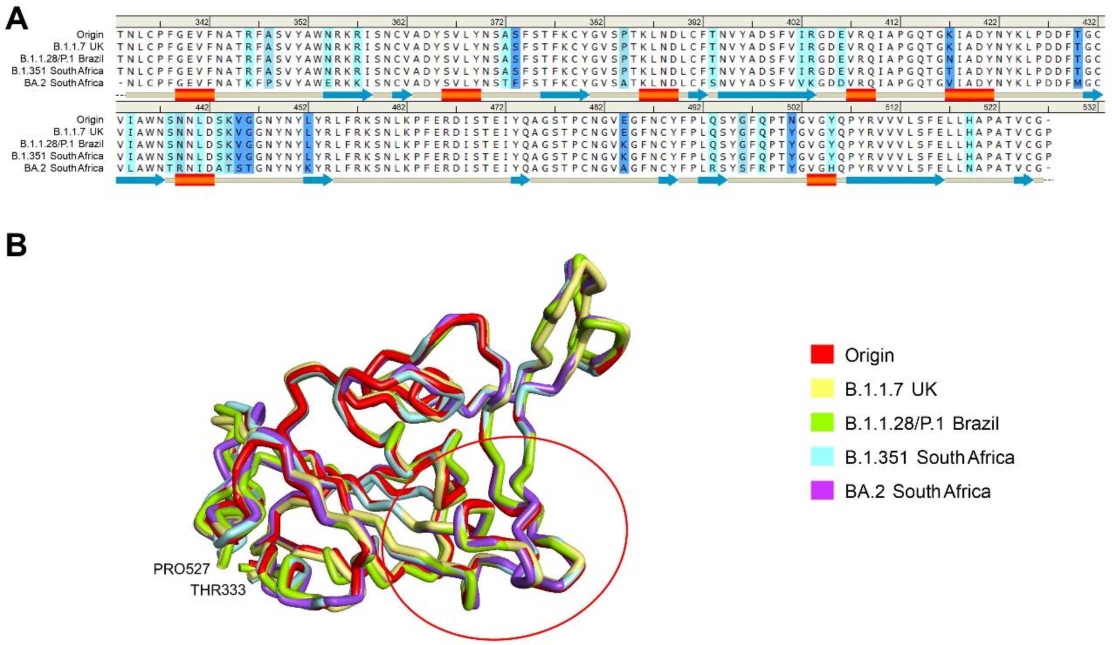

Figure S3. Overlap of SARS-CoV-2 RBD mutants. (**A**) Amino Acid Sequence of the common SARS-CoV-2 RBD mutants (B.1.1.7 UK, B.1.1.28/P.1 Brazil, B.1.351 South Africa and BA.2 South Africa variant). The blue block indicates the mutation. (**B**) The SARS-CoV-2 RBD mutant structures were illustrated by sticks. The red circle highlights interaction amino acids in these RBD proteins.

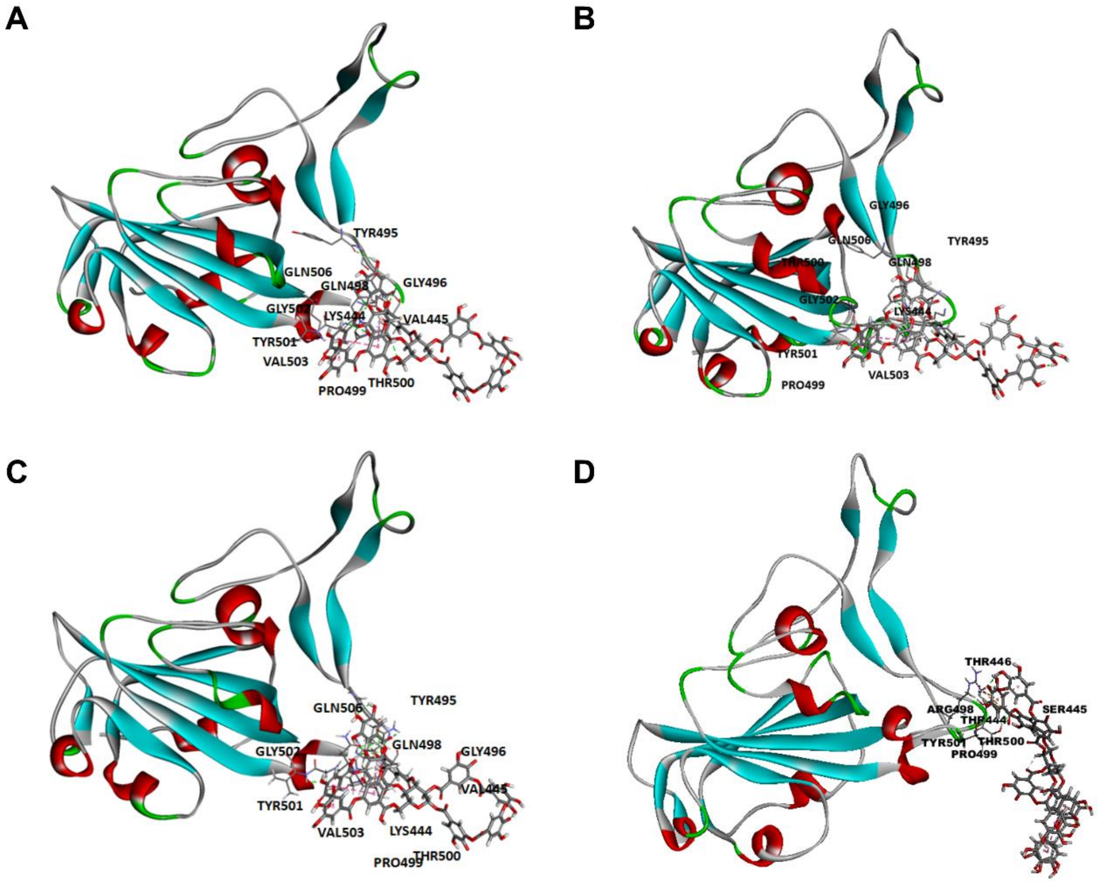

Figure S4. Tannic acid binding with typical mutants. (**A**) - (**D**) Docking analysis of tannic acid with common SARS-CoV-2 RBD mutants (B.1.1.7 UK, B.1.1.28/P.1 Brazil, B.1.351 South Africa and BA.2 South Africa variant). Tannic acid and key residues are shown as sticks with carbon, oxygen and nitrogen colored gray, red and blue, respectively. Electrostatic interaction was shown as dashed lines with π-π, π-alkyl, and hydrogen bonds colored purple, pink, and green, respectively. Secondary structural elements are depicted as ribbons (coils, α-helices; arrows, β-sheets). Color is based on secondary structures (α-helices, red; β-sheets, skyblue; loops, green)**.**

2 Supplementary Tables

Table S1. Mutation rate of the essential amino acids in the interaction of tannic acid SARS-CoV-2 RBD.

Genomic location	AA mutation	No. of AA with the mutation	AA mutation rate
22895	445V>L	5	4.84956E-07
	445V>I	164	1.59066E-05
	445V>F	186	1.80404E-05
22896	445V>G	7	6.78939E-07
	445V>D	5	4.84956E-07
	445V>A	310	3.00673E-05
22898	446G>C	2	1.93982E-07
	446G>R	194	1.88163E-05
	446G>S	729,833	0.07078739
22899	446G>A	66	6.40142E-06
	446G>D	163	1.58096E-05
	446G>V	6,585	0.000638687
23054	498Q>K	48	4.65558E-06
	498Q>E	25	2.42478E-06
23055	498Q>L	8	7.7593E-07
	498Q>R	1,143,900	0.110948252
	498Q>P	3	2.90974E-07
20356	498Q>H	43	4.17062E-06
23060	500T>A	26	2.52177E-06
	500T>P	6	5.81947E-07
	500T>S	67	6.49841E-06
23061	500T>I	20	1.93982E-06
	500T>N	11	1.0669E-06
	500T>S	35	3.39469E-06
23063	501N>D	18	1.74584E-06
	501N>H	46	4.4616E-06
	501N>Y	2,042,528	0.198107275
23064	501N>I	98	9.50514E-06
	501N>S	332	3.22011E-05
	501N>T	3,289	0.000319004
23065	501N>K	30	2.90974E-06

∗ AA, amino acids.

Table S2. Key amino acids of interactions.

Interaction	Type of interaction	Key amino acids
S-RBD and ACE2	Hydrogen bonds	Lys353, Asp38, Met82, Glu35, Lys31
	Hydrophobic interactions	Asn501, Gln493, Leu455, Ser494, Phe486
S-RBD and tannic acid	Hydrogen bonds	Gly446, Asn439, Thr500, Gln498
	Hydrophobic interactions	Lys444, Val445, Gly496, Phe497, Pro499, Gly502, Val503, Gln506, Pro507
ACE2 and tannic acid	Hydrogen bonds	Thr324, Phe327, Glu329, Arg357
	Hydrophobic interactions	Trp48, Asn330, Met332, Arg357
B.1.1.28 (Brazil) S-RBD and tannic acid	Hydrogen bonds	Gly446, Asn439, Thr500, Gln498
	Hydrophobic interactions	Lys444, Val445, Gly496, Phe497, Pro499, Gly502, Val503, Gln506, Pro507
B.1.1.7 (UK) S-RBD and tannic acid	Hydrogen bonds	Gly446, Asn439, Thr500, Gln498
	Hydrophobic interactions	Lys444, Val445, Gly496, Phe497, Pro499, Gly502, Val503, Gln506, Pro507
B.1.351 (South Africa) S-RBD and tannic acid	Hydrogen bonds	Gly446, Asn439, Thr500, Gln498
	Hydrophobic interactions	Lys444, Val445, Gly496, Phe497, Pro499, Gly502, Val503, Gln506, Pro507
BA.2 (South Africa) S-RBD and tannic acid	Hydrogen bonds	Thr444, Ser445, Thr446, Pro499, Thr500, Tyr501
	Electrostatic	Arg498
