# Supplementary figures and images for: Role of tannic acid against SARS-cov-2 cell entry by targeting the interface region between S-protein-RBD and human ACE2
Source: Front Pharmacol. 2022 Aug 8;13:940628. doi: 10.3389/fphar.2022.940628 (PMC9393390; doi:10.3389/fphar.2022.940628)

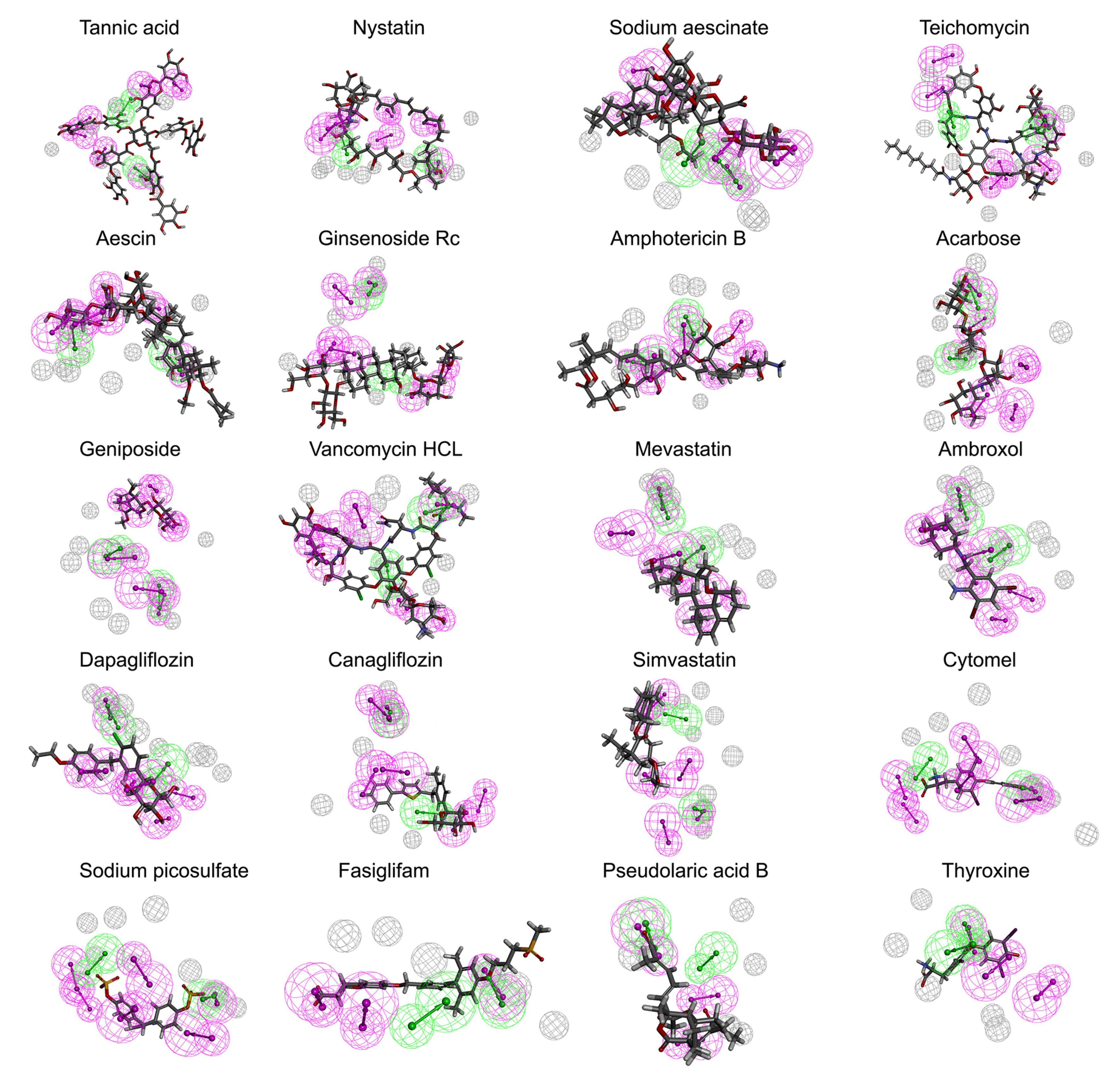

Supplement: Supplementary file 1 [file DataSheet1.ZIP › Figure S1.tif]

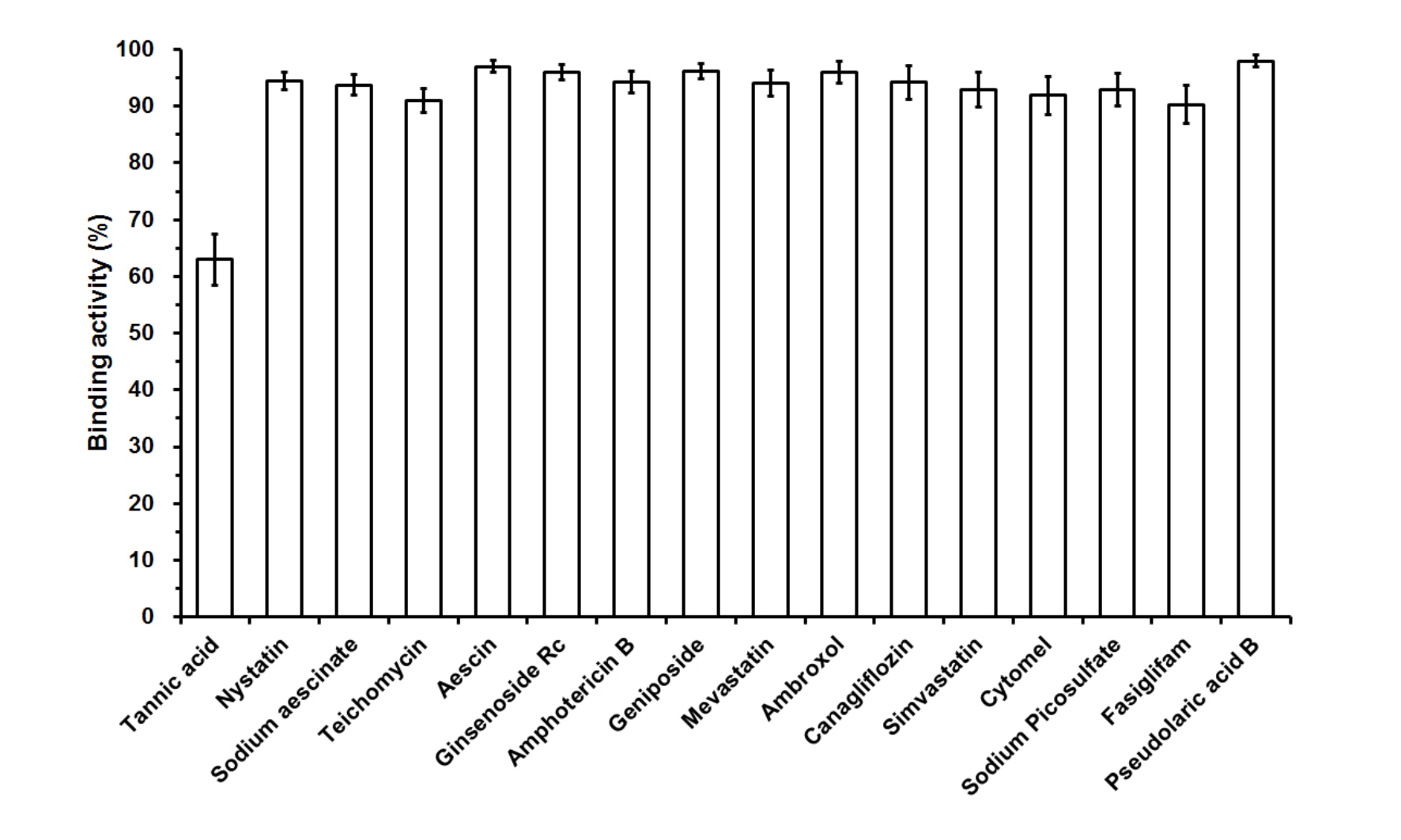

Supplement: Supplementary file 1 [file DataSheet1.ZIP › Figure S2.tif]

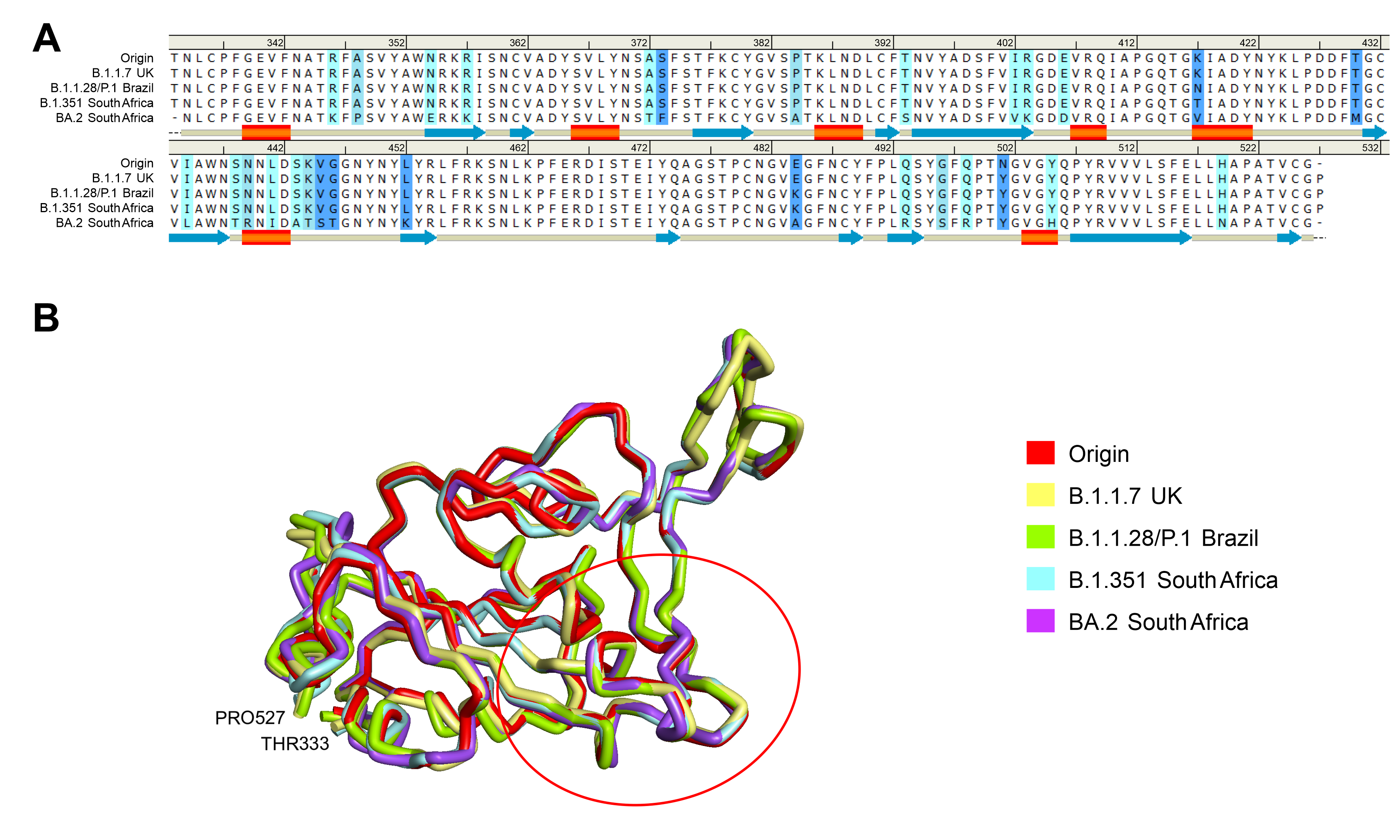

Supplement: Supplementary file 1 [file DataSheet1.ZIP › Figure S3.tif]

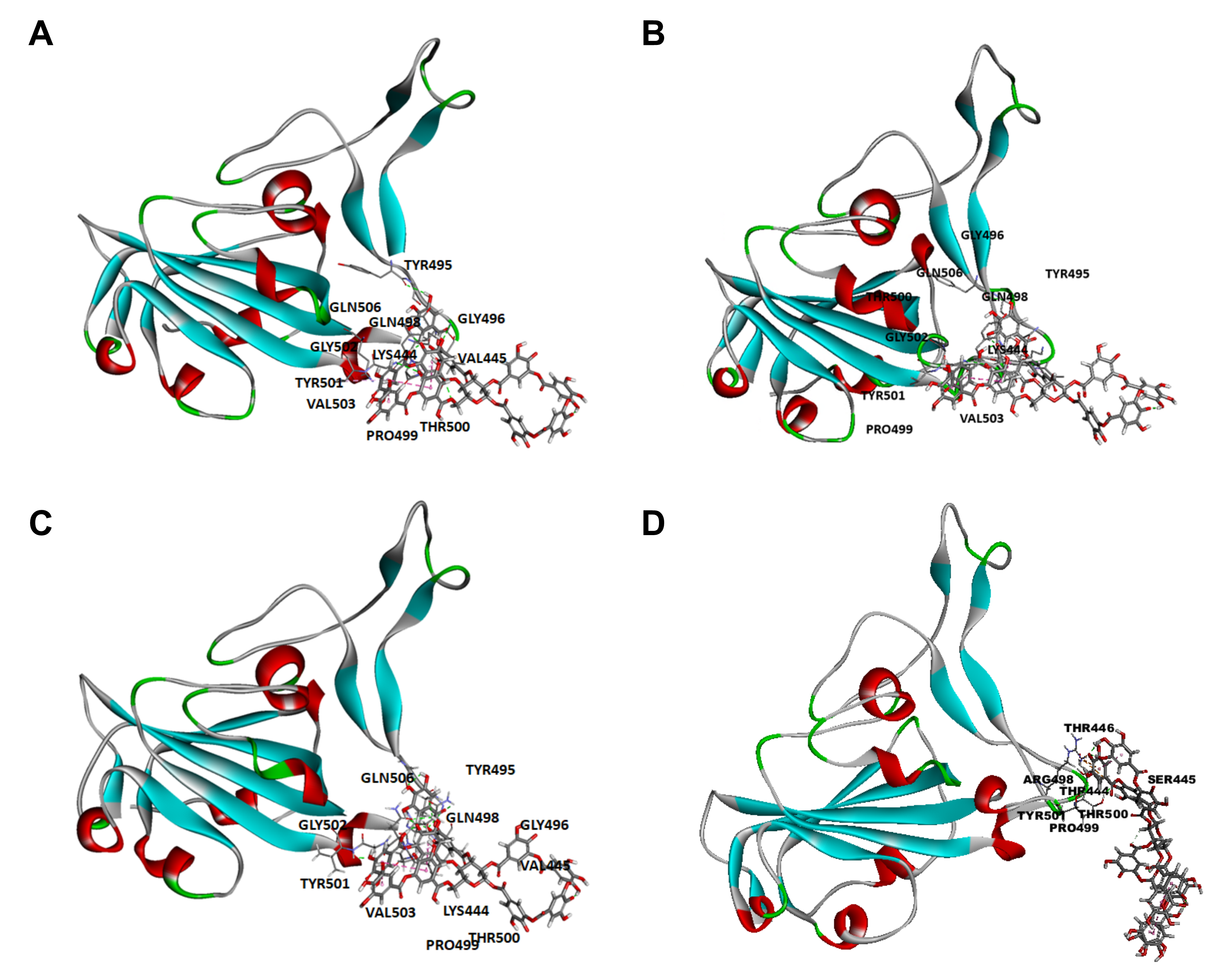

Supplement: Supplementary file 1 [file DataSheet1.ZIP › Figure S4.tif]
